# Long term outcome in children with shunted hydrocephalus

**Published:** 2012-11

**Authors:** Hosein Mashhadinezhad

**Affiliations:** ^a^Department of Neurosurgery, Mashhad University of Medical Sciences, Mashhad, Iran.

## Abstract

**Background:**

Hydrocephalus is a common disorder in pediatric neurosurgery. Notwithstanding recent improved therapeutic strategies for the management of pediatric hydrocephalus, there continues to be a significant mortality rate in children. The present study aims to determine the long-term survival and predictor factors of death in these patients.

**Methods:**

The data and recordings of pediatric hydrocephalus patients (under 2 years old) who underwent a cerebrospinal fluid (CSF) diverting shunt in the Department of Neurosurgery of Mashhad University of Medical Sciences (Iran), during 1993 to 2007 were recorded. Patients with neoplasms were excluded from further assessment. The patients were followed-up through clinical records or a family telephone call. The Kaplan-Meier survival estimates were used to determine overall survival of the patients. Univariate analysis was performed using the log-rank test. The multivariate analysis was performed by Cox's proportional hazard model to determine the significance of sex, age (at the time of initial shunt placement), shunt model and cause of hydrocephaly on patient survival. Furthermore, the hazard ratio with 95% confidence interval values was determined.

**Results:**

A total of 147 patients were participated in this study, of which 106 (72%) were available in the last follow- up, sixteen (15%) patients were died. The most common cause of death was infection (50%)%). The overall survival and mortality rate in all patients at 1, 5, and 10 years after initial shunt insertion to death or last follow- up visit was 96% , 91%, and 83% and 4%,9%, and 17%, respectively. Incidence of shunt-related failures was 46.2% in patients. There was no significant difference in survival with respect to sex, age of three months at the time of shunt insertion and shunt model. Cox’s regression model showed congenital hydrocephalic patients have more survival rate than myelomeningocele patients.

**Conclusions:**

This study showed the long-term survival rate in children with shunted hydrocephalus was 83%. Furthermore, the patients with congenital hydrocephalus showed better prognosis than other hydrocephalies. Myelomeningocele and infection were the most risk factors of death.

**Keywords:**

Pediatric hydrocephalus, Outcome, Risk factors, Myelomeningocele, Infection


					Volume 4, Suppl. 1 Nov 2012
Publisher: Kermanshah University of Medical Sciences
URL: http://www.jivresearch.org
Copyright:
Congress of Iranian Neurosurgeons, 14-16 November 2012, Kermanshah, Iran
Paper No. 1
Long term outcome in children with shunted hydrocephalus
Hosein Mashhadinezhad a,*
a
Department of Neurosurgery, Mashhad University of Medical Sciences, Mashhad, Iran.
Abstract:
Background: Hydrocephalus is a common disorder in pediatric neurosurgery. Notwithstanding recent improved
therapeutic strategies for the management of pediatric hydrocephalus, there continues to be a significant mortality
rate in children. The present study aims to determine the long-term survival and predictor factors of death in these
patients.
Methods: The data and recordings of pediatric hydrocephalus patients (under 2 years old) who underwent a
cerebrospinal fluid (CSF) diverting shunt in the Department of Neurosurgery of Mashhad University of Medical
Sciences (Iran), during 1993 to 2007 were recorded. Patients with neoplasms were excluded from further
assessment. The patients were followed-up through clinical records or a family telephone call. The Kaplan-Meier
survival estimates were used to determine overall survival of the patients. Univariate analysis was performed using
the log-rank test. The multivariate analysis was performed by Cox's proportional hazard model to determine the
significance of sex, age (at the time of initial shunt placement), shunt model and cause of hydrocephaly on patient
survival. Furthermore, the hazard ratio with 95% confidence interval values was determined.
Results: A total of 147 patients were participated in this study, of which 106 (72%) were available in the last
follow- up, sixteen (15%) patients were died. The most common cause of death was infection (50%)%). The overall
survival and mortality rate in all patients at 1, 5, and 10 years after initial shunt insertion to death or last follow- up
visit was 96% , 91%, and 83% and 4%,9%, and 17%, respectively. Incidence of shunt-related failures was 46.2%
in patients. There was no significant difference in survival with respect to sex, age of three months at the time of shunt
insertion and shunt model. Cox's regression model showed congenital hydrocephalic patients have more survival rate
than myelomeningocele patients.
Conclusion: This study showed the long-term survival rate in children with shunted hydrocephalus was 83%.
Furthermore, the patients with congenital hydrocephalus showed better prognosis than other hydrocephalies.
Myelomeningocele and infection were the most risk factors of death.
Keywords:
Pediatric hydrocephalus, Outcome, Risk factors, Myelomeningocele, Infection
* Corresponding Author at:
Hosein Mashhadinezhad: Associate professor of Neurosurgery, Department of Neurosurgery, Mashhad university of Medical Sciences, Mashhad, Iran.
Tel: +98 511 801 2613; fax: +98 511 841 3493, E-mail: mashhadinejadh@mums.ac.ir, (Mashhadinezahad H.).

				

